# Supplementary material for: Fabrication of Activated Carbon Decorated with ZnO Nanorod-Based Electrodes for Desalination of Brackish Water Using Capacitive Deionization Technology
Source: Int J Mol Sci. 2023 Jan 11;24(2):1409. doi: 10.3390/ijms24021409 (PMC9866127; doi:10.3390/ijms24021409)
Supplement: Supplementary file 1 [file ijms-24-01409-s001.zip › ijms-2053821-supplementary.pdf]

Supporting Information

Fabrication of activated carbon decorated with ZnO nanorods-based electrodes for desalination of brackish water using capacitive deionization technology.

Jhonatan Martinez, Martín Colán, Ronald Castillón, Pierre Ramos, Robert Paria, Luis Sánchez and Juan M. Rodríguez*

Center for the Development of Advanced Materials and Nanotechnology, Universidad Nacional de Ingeniería, Av. Túpac Amaru 210, Lima 15333, Peru

* Correspondence: jrodriguez@uni.edu.pe

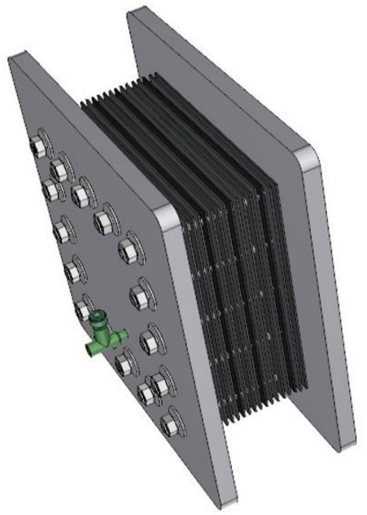

Figure S1. CDI cell fabricated with nine pairs of electrodes

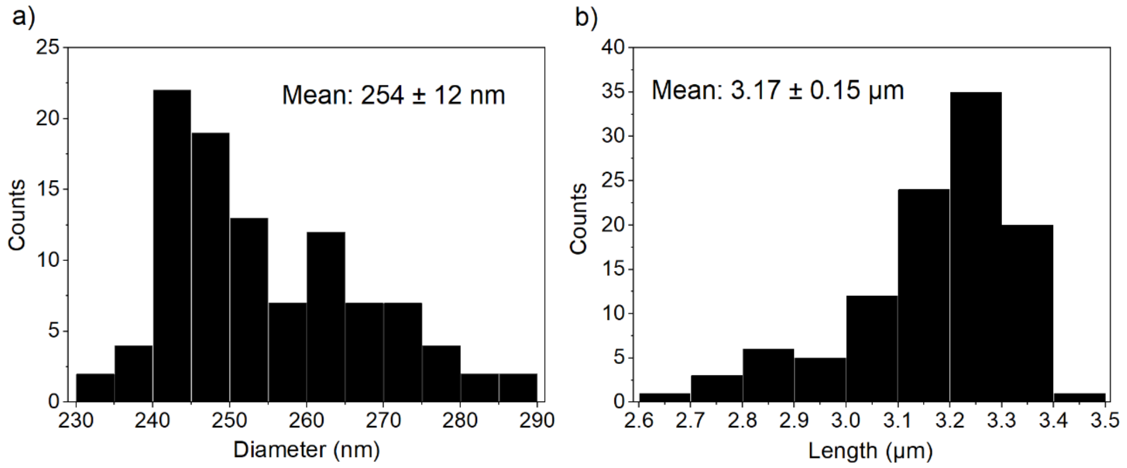

Figure S2. Histograms showing the distribution of measured a) Diameter and b) Length of ZnO nanostructures.

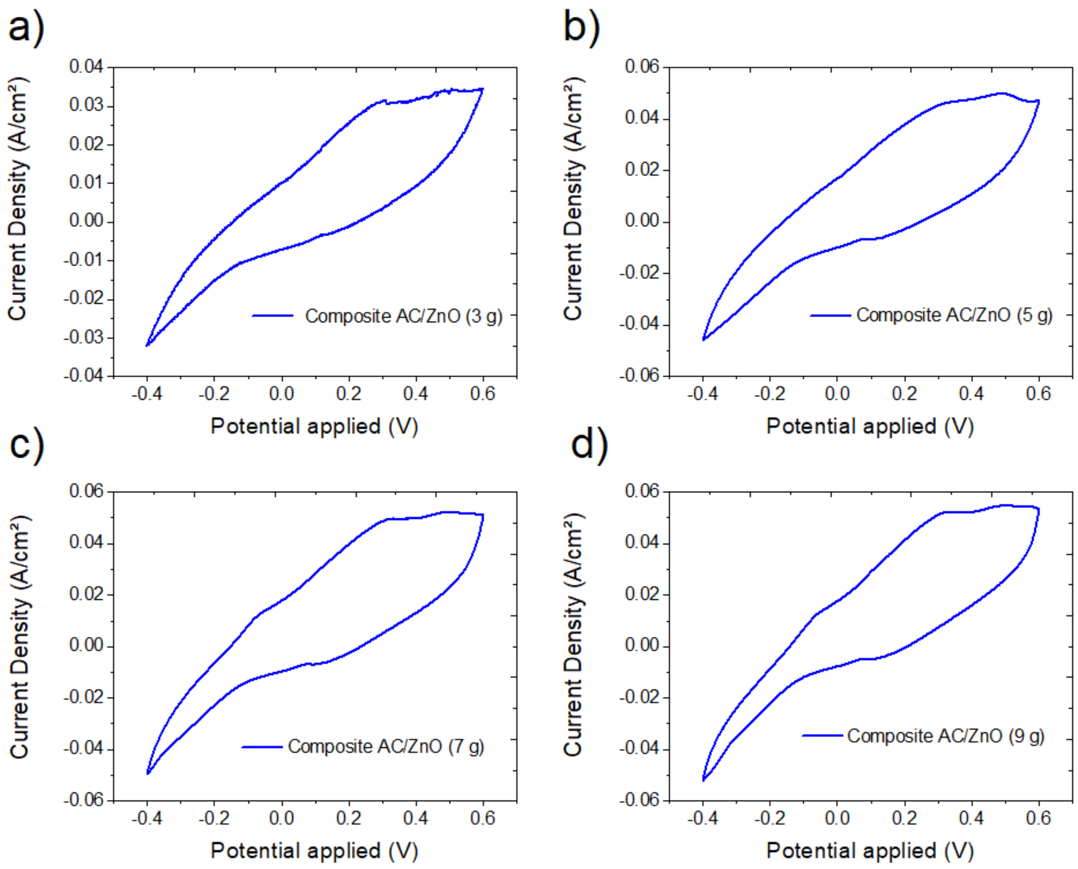

Figure S3. Cyclic voltammograms corresponding to the CA/ZnO composites for different masses of ZnO. a) 3 g of ZnO powder, b) 5g of ZnO powder, c) 7 g of ZnO powder and d) 9 g of ZnO powder.

Table S1. BET Specific Surface area: Activated carbon and Activated carbon/ZnO (9 g)

Specimen	BET Specific surface area (m^2/g)	Specific surface area mesopores (m^2/g)	Specific surface area micropores (m^2/g)	Pore size (nm)
Activated Carbon	33 ± 1	33 ± 1	Without presence	12,5
Activated Carbon/ZnO (9 g)	182 ± 1	124 ± 1	58 ± 1	8,1
